# Metavariables Resuming Host Immune Features and Nodal Involvement Are Associated with Oncological Outcomes in Oral Cavity Squamous Cell Carcinoma

**DOI:** 10.3390/cells10092203

**Published:** 2021-08-26

**Authors:** Francesco Missale, Mattia Bugatti, Davide Mattavelli, Silvia Lonardi, Davide Lombardi, Piero Nicolai, Cesare Piazza, Simonetta Battocchio, Anna Maria Bozzola, Stefano Calza, William Vermi

**Affiliations:** 1 Department of Molecular and Translational Medicine, University of Brescia, 25125 Brescia, Italy; 2 Department of Head & Neck Oncology & Surgery Otorhinolaryngology, Antoni Van Leeuwenhoek, Nederlands Kanker Instituut, 1066 Amsterdam, The Netherlands; 3 Unit of Pathology, ASST Spedali Civili di Brescia, 25100 Brescia, Italy; mattia.bugatti@unibs.it (M.B.); silvia.lona@gmail.com (S.L.); simonetta.battocchio@gmail.com (S.B.); anna.bozzola@gmail.com (A.M.B.); 4 Unit of Otorhinolaryngology—Head and Neck Surgery, Department of Medical and Surgical Specialties, Radiological Sciences, and Public Health, University of Brescia, 25123 Brescia, Italy; davide.mattavelli@unibs.it (D.M.); davinter@libero.it (D.L.); ceceplaza@libero.it (C.P.); 5 Section of Otorhinolaryngology—Head and Neck Surgery, Department of Neurosciences, University of Padua, Via Giustiniani, 2-35128 Padua, Italy; pieronicolai@icloud.com; 6 Unit of Biostatistics, Department of Molecular and Translational Medicine, University of Brescia, 25125 Brescia, Italy; stefano.calza@unibs.it; 7 BDbiomed, Big and Open Data Innovation Laboratory, University of Brescia, 25125 Brescia, Italy; 8 Department of Pathology and Immunology, Washington University School of Medicine, St. Louis, MO 63130, USA

**Keywords:** oral neoplasms, lymphocyte, CD8, biomarker, survival modeling, data reduction, PCA, TIL, peripheral, head and neck

## Abstract

Oral cavity squamous cell carcinoma (OSCC) is a common head and neck cancer characterized by a poor prognosis associated with locoregional or distant failure. Among the predictors of prognosis, a dense infiltration of adaptive immune cells is protective and associated with improved clinical outcomes. However, few tools are available to integrate immune contexture variables into clinical settings. By using digital microscopy analysis of a large retrospective OSCC cohort (n = 182), we explored the clinical significance of tumor-infiltrating CD8^+^ T-cells. To this end, CD8^+^ T-cells counts were combined with well-established clinical variables and peripheral blood immune cell parameters. Through variable clustering, five metavariables (MV) were obtained and included descriptors of nodal (NODAL^MV^) and primary tumor (TUMOR^MV^) involvement, the frequency of myeloid (MYELOID^MV^) or lymphoid (LYMPHOID^MV^) peripheral blood immune cell populations, and the density of tumor-infiltrating CD8^+^ T-cells (TI-CD8^MV^). The clinical relevance of the MV was evaluated in the multivariable survival models. The NODAL^MV^ was significantly associated with all tested outcomes (*p* < 0.001), the LYMPHOID^MV^ showed a significant association with the overall, disease-specific and distant recurrence-free survival (*p* < 0.05) and the MYELOID^MV^ with the locoregional control only (*p* < 0.001). Finally, TI-CD8^MV^ was associated with distant recurrence-free survival (*p* = 0.029). Notably, the performance in terms of survival prediction of the combined effect of NODAL^MV^ and immune metavariables (LYMPHOID^MV^, MYELOID^MV^ and TI-CD8^MV^) was superior to the TNM stage for most of the outcomes analyzed. These findings indicate that the analysis of the baseline host immune features are promising tools to complement clinical features, in stratifying the risk of recurrences.

## 1. Introduction

Oral cavity squamous cell carcinoma (OSCC) is one of the most frequent head and neck tumors [1] with a rising incidence in the Western countries [2,3]. The clinical behavior of OSCC is characterized by the occurrence of early lymphatic spreading to regional lymph nodes. Among clinical and pathological features, nodal involvement is per se one of the most relevant prognostic factors [4,5], with an upstage to Stage III–IV and 42–43% 5-year estimated overall survival [5,6]. Furthermore, despite achieving early diagnosis, OSCC is characterized by a poor prognosis when locoregional or distant failure occurs [7], thus including the mandatory management of the neck with elective node dissection [8] or sentinel node biopsy [9] also for low-stage tumors. The main treatment modality for naïve tumors is still represented by radical surgery retaining radiotherapy (RT) or cisplatin-based chemo-radiotherapy (CT-RT) as adjuvant treatments.

The spectrum of available treatment options in the metastatic/recurrent not resectable setting, for which the median survival ranges between 6 and 15 months, is limited to conventional cytotoxic therapy (platinum-based chemotherapy, fluorouracil and taxanes), molecular target agents as anti-EGFR monoclonal antibodies and immunotherapy with PD-L1/PD-1 checkpoint inhibitors. The results obtained with the CheckMate 141, the KEYNOTE-012, and the KEYNOTE-048 trials established the role and use of pembrolizumab or nivolumab (PD-1 checkpoint inhibitors) with or without chemotherapy as first-line therapy in this clinical scenario [10,11,12]. The evaluation of the PD-L1 expression on tumor cells and tumor infiltrating cells, in the so-called CPS score [13], nowadays represent the key biomarker for the choice of systemic therapy modality [14]. However, its efficacy in patients’ selection is still debated. Several prognostic and predictive biomarkers have been recently proposed and tested in a wide retrospective cohort of OSCC patients, including a measure of the immune contexture on tissue slides. In addition to novel histological features [15,16] and morphology-based immune contexture parameters [17], recent meta-analysis showed that, in OSCC, a favorable overall outcome is associated with a high density of tumor infiltrating lymphocytes (TIL) as NK-cells, CD45RO^+^ T-cells and CD8^+^ T-cells, mainly if measured in the tumoral site [18,19], whereas tumor-associated CD68^+^ or CD163^+^ macrophages predict a worse prognosis [19]. The routine availability of pre-treatment blood samples raised the interest of studying circulating biomarkers. Among these, the neutrophil, lymphocyte, monocyte or platelet counts and the derived ratios have shown prognostic relevance among solid tumors with myeloid predominance, mainly associated with the worse outcomes [20,21,22]. One of the main limitations of the available literature is the paucity of analysis considering specific oncologic outcomes, including the locoregional or distant failures separately. For biomarkers analysis, the identification of cut-offs is still a matter of debate [23], likely overcome by avoiding dichotomization and keeping the whole information of a continuous variable [24]. Moreover, few attempts have been made to combine peripheral and tumoral immune-features in a unique classifier [25].

Within a homogeneous cohort of surgically treated OSCCs, we combined peripheral and tissue immune features with demographic, clinical and pathological characteristics to generate meta-variables (MVs). The MV describing the nodal involvement was confirmed to be detrimental for any survival end-points, whereas the peripheral blood myeloid-related MV and the CD8^+^ T-cells infiltration MV were significant predictors of locoregional or distant failure, respectively.

## 2. Materials and Methods

### 2.1. Clinical Cohort

A retrospective observational study was carried out, enrolling one hundred eighty-two histologically confirmed cases of oral cavity squamous cell carcinoma (OSCC) who underwent radical surgical resection and neck dissection between 2000 and 2014 (Otorhinolaryngology Department, ASST Spedali Civili di Brescia, Brescia, Italy). This study was approved by the local IRB to WV (H&N Cancer, NP-2066). Patients with at least 12 months of follow-up or earlier death or recurrence were included. Salvage surgery, metastatic disease, immunological disorders or prior systemic treatment for malignancy represented the exclusion criteria. Patients were regularly followed up with clinical examinations and neck MRI or CT every 3–6 months. Preliminary clinical and oncological findings are reported in our previous works [26,27]. Last follow-up was updated until September 2020.

### 2.2. Blood Samples

Preoperative blood cell counts were retrieved. The absolute counts of full white blood cells (WBC), neutrophils, lymphocytes, monocytes and platelets were considered as the biomarkers of interest. Further derived parameters of clinical interest according to the recent literature, the neutrophil-to-lymphocyte ratio (NLR) [22,28], i.e., the ratio between neutrophils and lymphocytes counts and the platelet-to-lymphocyte ratio (PLR) [29], i.e., the ratio between platelets and lymphocytes counts were obtained.

### 2.3. Tissues

Formalin-fixed paraffin embedded (FFPE) tissue blocks of a representative section of the primary tumor (PT) were retrieved from the tissue bank of the Department of Pathology (ASST Spedali Civili di Brescia, Brescia, Italy). Four-micron thick FFPE sections were used for immunohistochemical staining and as a primary antibody, the anti-CD8 (clone C8/144B, dilution 1:30, Dako) was used. The reaction was revealed by EnVision (Dako) followed by DAB. Sections were then counterstained with hematoxylin.

### 2.4. Digital Pathology Analysis

**Figure 1:**
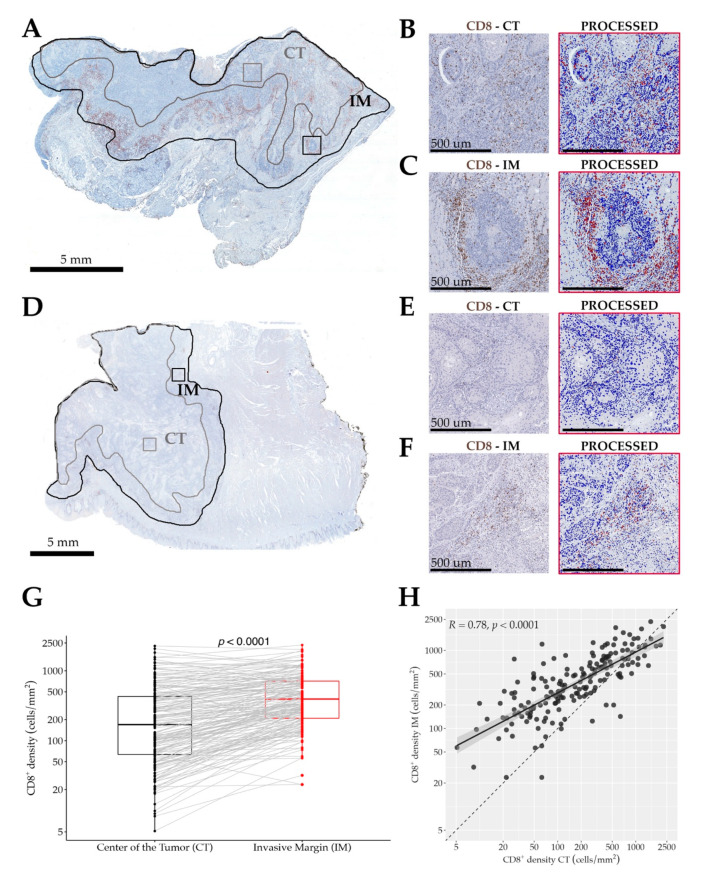
Panel showing whole tumor sections stained for CD8 in two representative cases (**A**–**C** and **D**–**F**). In (**A**) and (**D**)**,** the selection of areas of interest is shown: the center of the tumor (CT) and the invasive margin (IM)—whilst the black line defines the whole tumoral bed. Representative high magnification immunostained (**left**) and processed (**right**) sections showing the immune cells recognition and counting algorithm (**B**,**C**,**E**,**F**). In (**B**) and (**C**)**,** the example fields of 1 mm^2^ of the case presented in (**A**) taken from the CT (554 CD8^+^ T-cells) or IM (1159 CD8^+^ T-cells), respectively. In (**E**) and (**F**)**,** the example fields of 1 mm^2^ of the case presented in (**D**) are taken from the CT (26 CD8^+^ T-cells) or IM (294 CD8^+^ T-cells), respectively. The box plots (**G**) show a higher density of CD8^+^ T-cells in the IM compared to the CT (grey lines connecting the measures of CD8^+^ T-cell density in each single patient) and the scatter plot (**H**) illustrates a significant direct correlation of CD8^+^ T-cells’ density between those regions (**H**); p values estimated by the Wilcoxon matched-pairs signed rank test (**G**) or Spearman rank correlation test (**H**). Scale bars: (**A**,**D**), 5 mm; (**B**,**C**,**E**,**F**), 500 µm.

Stained slides were acquired using a ScanScope CS (Leica Microsystems, Wetzlar, Germany) digital scanner. Images were viewed and organized using ImageScope software (version 12.03.5048, Leica biosystems, Wetzlar, Germany). Each scanned image was manually annotated and the IHC nuclear image analysis algorithm was chosen for the analysis. Data are expressed as the number of CD8^+^ cells per mm^2^. Primary tumor (PT) was analyzed measuring the immune cell density either in the center of the tumor (CT) and in the invasive margin (IM) (Figure 1). The invasive margin was defined as the tissue area of 1 mm wide from the front of invasion of the tumor [30].

### 2.5. Statistical Analysis

The main goal was to summarize all the available clinical and biomarkers data in a minimal number of synthetic components, each representing a set of highly correlated variables, therefore identifying a few homogeneous latent variables. Applying a data reduction method allows the multivariable survival modelling of cohorts with a limited sample size and event rates [31].

Firstly, clinical, pathological and biomarkers variables were clustered using hierarchical clustering with a bottom-up agglomerative algorithm. This algorithm aimed to maximize the clusters’ homogeneity, defined as the proximity of each variable in the cluster to a synthetic centroid, using as proximity metrics either the Pearson correlation coefficient for quantitative variables or the correlation ratio for qualitative ones [32]. The optimal number of partitions was determined evaluating clusters’ stability measured by the Rand index between the observed hierarchy and a random sample of dendrograms generated by bootstrap (B = 200). Finally, a synthetic variable representing each cluster was computed as the first principal component derived by a mixed principal component analysis (PCAmix) performed on the variable within the cluster [32,33].

Quantitative variables were summarized using the mean, standard deviation, median and range, while qualitative variables were described as counts and proportions. For group comparisons in qualitative variables, Fisher’s exact test was applied, and for quantitative ones, the Wilcoxon test or Kruskal-Wallis test was applied. Missing data, representing 2.6% of the measured data, were imputed with multivariate imputation by chained equations (MICEs) [34].

The survival endpoints considered are the overall (OS), the disease-specific (DSS), the locoregional recurrence-free survival (LRFS), and the distant recurrence-free (DRFS) survival. The OS was defined as the time between the date of surgery and the date of death for any causes; the DSS as the time between the date of surgery and the date of cancer-related death; the LRFS as the time between the date of surgery and the date of local or nodal recurrence; and the DRFS as the time between the date of surgery and the date of distant recurrence. For each outcome, patients now having the event were censored at the last follow-up.

Survival analysis was performed fitting multivariable Cox proportional-hazards models, estimating *p* values by the Wald statistic. The relationship between continuous predictor and the outcomes was modelled with restricted cubic splines with 4 knots [35]; not linear terms were retained in the presented models for variables mostly explaining the X^2^ statistic [36]. The likelihood ratio test was applied for comparing nested models. Decision curve analysis (DCA) [37,38,39] was used to evaluate the net benefit of each proposed model compared to the one fitted considering the UICC overall pathological stage alone. Survival estimates were reported as hazard ratios (HR) with 95% confidence interval (95% CI) and estimating the 2- and 5-year survival probability with 95% CI for the variables of main clinical interest. Proportional hazards assumption was tested examining Schoenfeld residuals [40]. Data reduction analysis was performed with the ‘ClustOfVar’ package [32]. Contour plots were drawn with the ‘visreg’ package [41] and DCA was performed with the ‘dcurves’ one. In all analyses, two-tail tests with a significance level of 5% were applied; adjusted *p* values for multiple tests were corrected with Bonferroni’s method. R version 4.1.0 (R Foundation for Statistical Computing, Vienna, Austria) was used for statistical analysis.

## 3. Results

### 3.1. Clinical Findings of the OSCC Cohort

One hundred and eighty-two patients were enrolled for data analysis; the cohort was composed of 115 males (63.2%) with a mean ± SD age of 63.6 ± 13.1 years. Among the clinical, pathological and biomarker variables of interest, missing data accounted for 2.6% of the dataset, with no variable with “missingness” ≥10% (Supplementary Figure S1 and Supplementary Table S1). Considering already imputed data (Table 1), the cohort was well balanced among all pT categories, and metastatic lymph-node involvement was recorded in 85 cases (46.7%), and in 42 cases (23.1%), evidence of pathologic extranodal extension was observed. The mean ± SD number of involved positive nodes was 1.54 ± 2.84, ranging from 0 to 18. Adverse pathological features as perineural invasion (PNI) or lymphovascular invasion (LVI) were present in 90 (49.5%) and 52 (28.6%) cases, respectively. The rate of positive margin was 17.6%. The treatments included surgery alone in 78 patients (42.9%), surgery and adjuvant radiotherapy (RT) in 61 (33.5%), and surgery followed by adjuvant chemo-radiotherapy (CRT) in 43 (23.6%). 

The mean follow-up time was 71.5 months (IQR 24.3–105.8, range 1–214 months). During the follow-up course, 71 patients (39.0%) experienced at least one recurrence event, 42 (23.1%) experienced locoregional recurrence alone, 14 (7.7%) distant recurrence alone and 15 (8.2%) both locoregional and distant recurrences. At the last follow-up available, 78 patients (42.9%) were dead and for 53 of these (67.9%), the cause of death was related to disease progression. 

### 3.2. Clinical Relevance of CD8 Immune Contexture and Peripheral Blood Biomarkers in OSCC

By digital image analysis, the CD8^+^ T-cell infiltration of tumor tissue was evaluated by measuring a mean ± SD area of 108.7 ± 73 mm^2^ (range 4.2–319.9 mm^2^). Analyzing both the center of the tumor (CT) and the invasive margin (IM), the latter was significantly enriched (*p* < 0.0001) of CD8^+^ T-cells (median 394 cells/mm^2^, IQR 210–712) compared to the CT (median 170 cells/mm^2^, IQR 64–430), as shown in Figure 1G; furthermore, a strong direct correlation between the density of CD8 T-cells in CT and IM was evident (R = 0.78 (CI_95%_ 0.71–0.83, *p* < 0.0001, Figure 1H)); representative fields of the analysis are shown in Figure 1**.** Whilst testing for associations and correlations., a lower ^IM^CD8^+^ T-cells density was observed in patients with evidence of LVI (*p* = 0.032); no other meaningful association with further clinical or pathological features was observed (Supplementary Tables S1–S9). Finally, no correlation was observed between intratumoral CD8^+^ T-cells density and any of the available peripheral blood biomarkers (Supplementary Table S1).

Analyzing the available pre-operative peripheral blood biomarkers, a significant association was observed, as expected, between the sex and all the absolute biomarkers and lymphocyte counts (Supplementary Table S2). Furthermore, no relevant associations or correlations were observed between such measures and the other clinical and pathological features (Supplementary Tables S1–S9).

### 3.3. Identification of the Meta-Variables in OSCC

**Figure 2:**
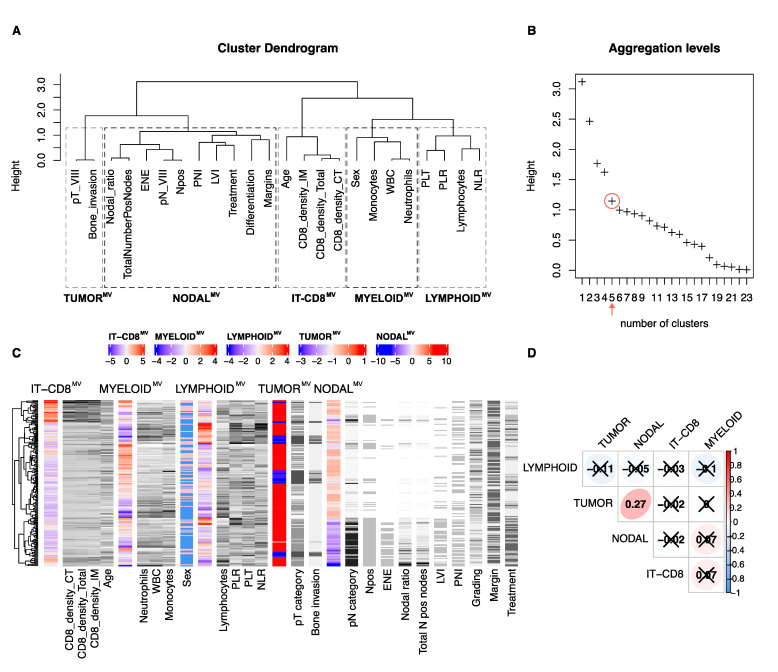
Dendrogram of the hierarchical ascending clustering of the 24 demographic, clinical, pathological and peripheral and tissue biomarkers according to their correlation ratio (**A**); aggregation level plot related to the number of clusters of variables, red circle showing the chosen 5 clusters as optimum choice (**B**); heatmap showing the relationship between each metavariable (MV) and the original variables from which is derived (columns on the right of each metavariable) (**C**); and correlogram showing the Spearman correlation between each metavariable, R coefficient is shown, adjusted not significant comparisons are covered with a cross (**D**). Dashed rectangles in A highlight which original variable is included in each MV.

With the aim of summarizing all available clinical and biomarkers variables in a minimal number of synthetic components, a data reduction algorithm was applied [32,33]. The available 24 variables were resumed into metavariables (MV). The aggregation of highly correlated variable was obtained by ascending hierarchical clustering, (Figure 2A). By inspecting the scree plot (Figure 2B), five clusters were identified, from here on referred to as metavariables (MVs). Specifically, the identified MV are the IT-CD8^MV^, MYELOID^MV^, LYMPHOID^MV^, TUMORAL^MV^ and NODAL^MV^, whose composition is highlighted by dashed rectangles in Figure 2A. The relationship between the MV scores and the variables is shown in Figure 2C and summarized in Supplementary Figure S2–S6; full details of the squared loadings (as a measure of the weight of each variable within the MV) are reported in Supplementary Table S10. MVs did not show significant correlations, except for a slight direct correlation (R = 0.27, *p* = 0.002) between the TUMORAL^MV^ and NODAL^MV^ (Figure 2D), confirming the appropriate segregation of the associated variables.

### 3.4. NODAL^MV^ Is Highly Related to OS and DSS

**Figure 3:**
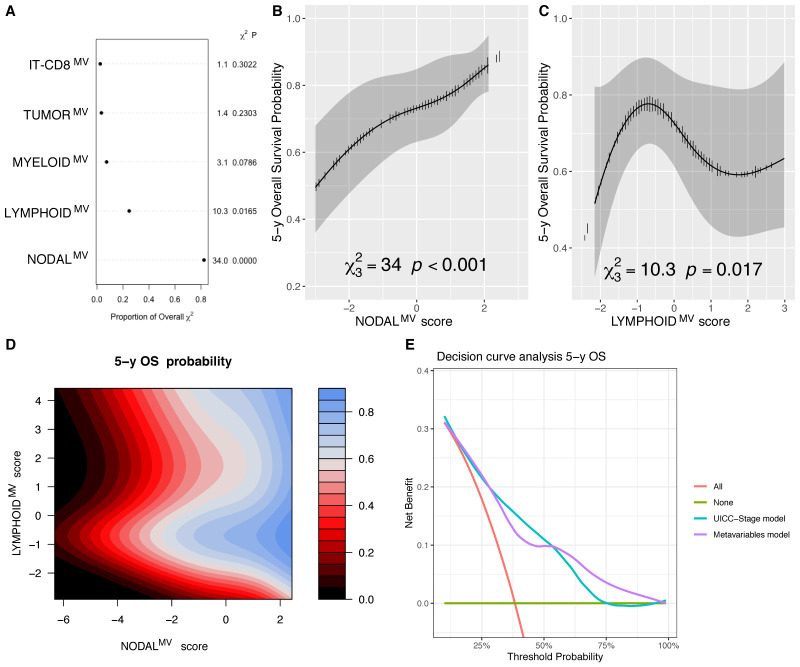
Chunk test results on the OS multivariable model reporting the proportion of the overall χ^2^ of the model explained by each variable, the partial χ^2^ and P value of the Wald test, testing the association between each variable and the outcome (**A**); adjusted marginal effect plot of the NODAL^MV^ score for the 5-year OS estimate with the CI_95%_ gray band (**B**); adjusted marginal effect plot of the LYMPHOID^MV^ score for the 5-year OS estimate with CI_95%_ gray band (**C**); contour plot showing isoprognostic OS bands according to the combined effect of the NODAL^MV^ and LYMPHOID^MV^ scores for the 5-year OS estimate, the color scale represents the 5 y OS probability (**D**); and decision curve analysis (DCA) analysis showing comparable results of the model including metavariables and to the one fitted with UICC overall stage alone in predicting the 5-year OS (**E**).

The NODAL^MV^, whose lower values (Supplementary Figure S2) are correlated with a high nodal category, high nodal burden (both lymph node ratio and number of positive nodes), presence of pathologic risk factors as ENE, PNI, LVI or high grade and positive margins was highly associated with all survival endpoints. NODAL^MV^ and the LYMPHOID^MV^ were significantly associated (*p* < 0.0001 and *p* = 0.0165, respectively) with OS (Figure 3A). Specifically, NODAL^MV^ showed a linear relationship (Figure 3B), whereas LYMPHOID^MV^ displayed a non-linear effect (Figure 3C) with the best outcome at its mid-values (Supplementary Table S11). The contour plot in Figure 3D represents isoprognostic areas according to the combined value of NODAL^MV^ and LYMPHOID ^MV^. Applying the likelihood ratio test, we found that the removal of the LYMPHOID^MV^ from the model significantly reduced its accuracy (*p* = 0.028), further supporting the relevance of this variable in the OS outcome. By decision curve analysis (DCA), the model including MVs was comparable to the UICC-TNM pathological Stage in terms of 5 years OS prediction (Figure 3E).

**Figure 4:**
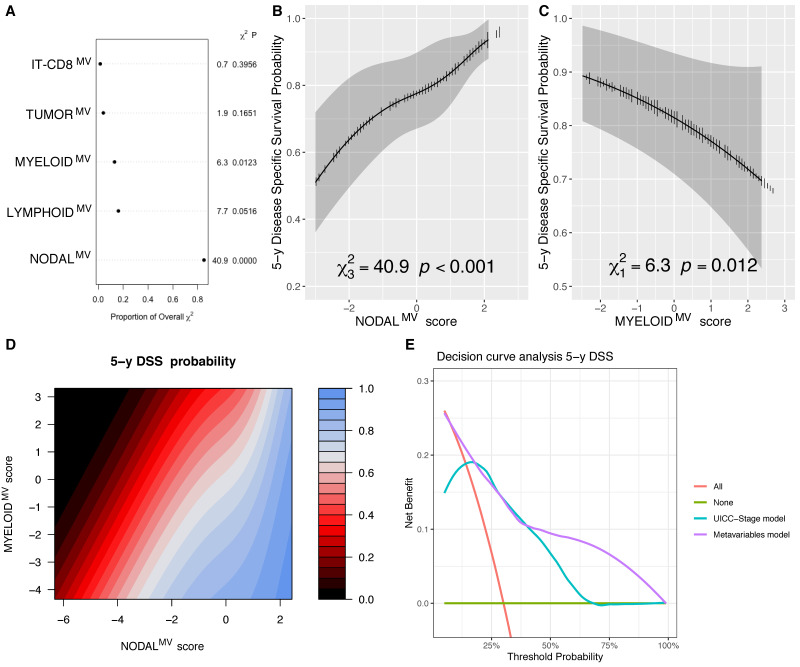
Chunk test results on the DSS multivariable model reporting the proportion of the overall χ^2^ of the model explained by each variable, the partial χ^2^ and *p* value of the Wald test, testing the association between each variable and the outcome (**A**); adjusted marginal effect plots for the 5-year DSS with CI_95%_ (gray bands) of the NODAL^MV^ (**B**) and MYELOID^MV^ (**C**) scores; contour plot showing isoprognostic DSS bands according to the combined effect of the NODAL^MV^ and MYELOID^MV^ scores for the 5-year DSS estimate, the color scale represents the 5 y DSS probability (**D**); and decision curve analysis (DCA) showing the advantages of the model including MV compared to the one fitted with UICC overall stage alone in predicting the 5-year DSS (**E**).

Analyzing the DSS, the NODAL^MV^ and MYELOID^MV^ were significantly associated with the outcome (*p* < 0.0001 and *p* = 0.0123, respectively; Figure 4A–C, Supplementary Table S11). Specifically, for MYELOID^MV^, higher scores (associated with leukopenia, neutropenia, monocytopenia and female sex) were associated with a poorer DSS, as shown in Figure 4C. The combined partial effect of NODAL^MV^ and MYELOID^MV^ on the 5-year DSS estimate is shown with the contour plot in Figure 4D that illustrates the detrimental contribution of different MYELOID^MV^ scores along the different values of NODAL^MV^ scores. By using the likelihood ratio test, the removal of the MYELOID^MV^ from the model significantly reduced its accuracy (*p* = 0.011), further supporting the relevance of such a variable for the DSS outcome. By DCA, the model including MVs showed a benefit in terms of the prediction of the 5-year DSS compared to the UICC-TNM pathological stage, (Figure 4E).

### 3.5. Immune MV as Predictor of Loco-Regional and Distant Failure

**Figure 5:**
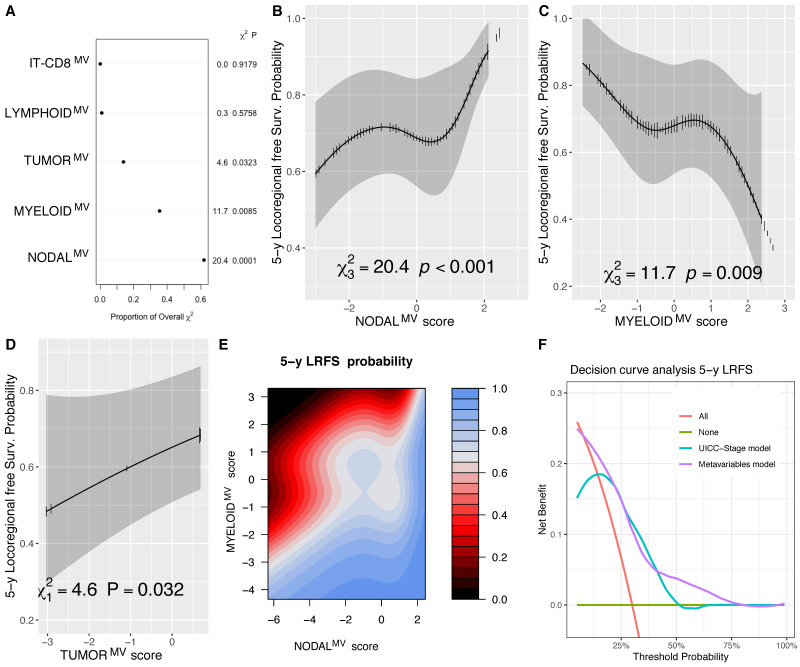
Chunk test results on the LRFS multivariable model reporting the proportion of the overall χ^2^ of the model explained by each variable, the partial χ^2^ and P value of the Wald test, testing the association between each variable and the outcome (**A**); adjusted marginal effect plots for the 5-year LRFS with CI_95%_ (gray bands) of the NODAL^MV^ (**B**); MYELOID^MV^ (**C**), and TUMOR^MV^ (**D**) scores; contour plot showing isoprognostic LRFS bands according to the combined effect of the NODAL^MV^ and MYELOID^MV^ scores for the 5-year LRFS estimate—the color scale representing the 5 y LRFS probability (**E**); and decision curve analysis (DCA) showing a better performance of the model including MVs compared to the one fitted with the UICC overall stage alone in predicting the 5-year LRFS in the lowest and highest ranges of the estimated risks (**F**).

During the follow-up course, 57 patients (31.3%) developed locoregional recurrence with a 5-year LRFS estimate of 78% (CI_95%_ 72–84%) and 29 distant metastases (15.9%), with an estimate of DRFS at 5 years of 85% (CI_95%_ 79–90%). Modelling the LRFS outcome, we could confirm the detrimental prognostic significance of the NODAL^MV^ (*p* < 0.001), MYLEOID^MV^ (*p* = 0.009) and TUMOR^MV^ (*p* = 0.032) (Figure 5A–D, Supplementary Table S11). The contour plot shown in Figure 5E illustrates the combined effect of NODAL^MV^ and MYELOID^MV^ scores for the 5-year LRFS prediction with iso-prognostic levels (color gradient) strictly dependent on both variables. By the likelihood ratio test, the removal of the MYELOID^MV^ from the model significantly reduced its accuracy (*p* = 0.004), further supporting the relevance of this variable for the LRFS outcome. By DCA and compared to the UICC-TNM pathological stage, the model including MVs showed a benefit in terms of predicting the 5-year LRFS, specifically in the lowest and highest ranges of the estimated risks (Figure 5F).

**Figure 6:**
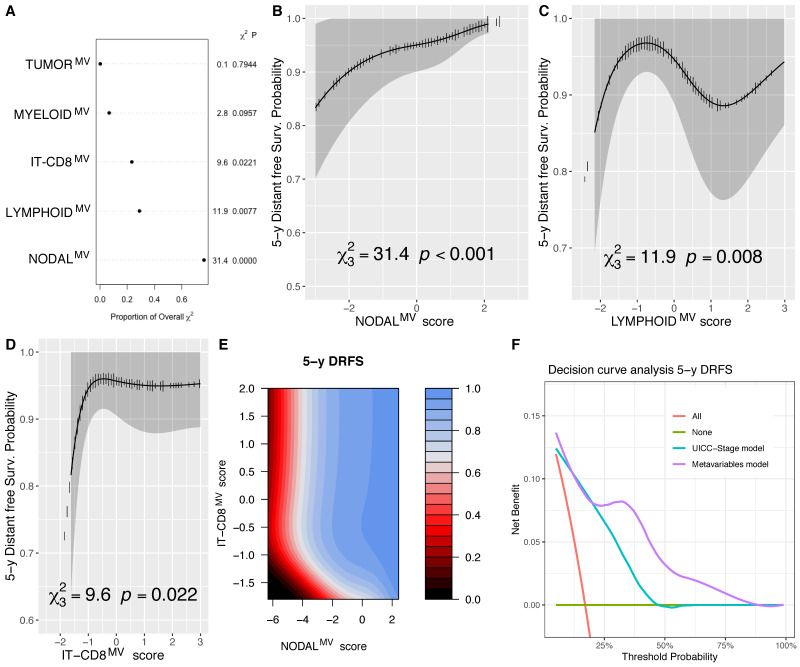
Chunk test results on the DRFS multivariable model reporting the proportion of the overall χ^2^ of the model explained by each variable, the partial χ^2^ and P value of the Wald test, testing the association between each variable and the outcome (**A**); adjusted marginal effect plots for the 5-year DRFS with CI_95%_ (gray bands) of the NODAL^MV^ (**B**); LYMPHOID^MV^ (**C**) and IT-CD8^MV^ (**D**) scores; contour plot showing isoprognostic DRFS bands according to the combined effect of the NODAL^MV^ and IT-CD8^MV^ scores for the 5-year DRFS estimate—the color scale represents the 5 y DRFS probability (**E**); and decision curve analysis (DCA) showing the better performance of the model including MVs compared to the one fitted with the UICC overall stage alone in predicting the 5-year DRFS (**F**).

Considering the DRFS outcome and its modeling with a multivariable model (Figure 6A, Supplementary Table S11), NODAL^MV^ confirmed its high weight for its association with such an outcome (*p* < 0.001, Figure 6B), furthermore LYMPHOID^MV^ (*p* = 0.008, Figure 6C) and IT-CD8^MV^ (*p* = 0.022, Figure 6D) were associated with DRFS following non-linear effects (*p* = 0.0037, *p* = 0.0537, respectively). The lowest IT-CD8^MV^ score, corresponding to low CD8 infiltration in all the tested areas of interest, were strictly related to a poor DRFS, also independently from the NODAL^MV^ score, as shown in the contour plot in Figure 6E. By using the Likelihood ratio test, the removal of IT-CD8^MV^ and LYMPHOID^MV^ from the model significantly reduced its accuracy (*p* = 0.036 and *p* = 0.013, respectively), further supporting the relevance of such variables for the DRFS outcome. By DCA, the model including MVs showed a benefit in terms of the prediction of the 5-year DSS, compared to the UICC-TNM pathological stage, shown in (Figure 6F).

## 4. Discussion

In this study, we tested the clinical significance of peripheral blood and tumor-associated immune features in patients submitted to surgery-based treatments for OSCC. 

Through a data reduction analysis, five metavariables (MV) were defined; the survival analysis showed their association with different types of oncological outcomes. Specifically, the NODAL^MV^ was independently associated with all oncological outcomes tested, thus confirming the detrimental prognostic value of nodal involvement in OSCC. Information derived from peripheral blood biomarkers, and resumed in the MYELOID^MV^ and LYMPHOID^MV^, significantly improved the accuracy of the models of DSS/LRFS and of OS/DRFS, respectively. Thus, these MV represent candidates for the development of predictors models. Interestingly, the IT-CD8^MV^ was significantly associated with the DRFS, suggesting that the group of OSCC desert of CD8^+^ T-cells should be deeply investigated to identify an innovative therapeutical strategy limiting distant spread. Furthermore, the proposed models including MVs outperform the UICC TNM stage in predicting most of the outcome analyzed.

It is well-known that several clinical variables (e.g., features describing the nodal involvement such as N category, number of positive nodes, LNR, presence of ENE, or high-risk features as PNI, LVI or grading and T category and bone involvement) are highly correlated and it is often difficult to select which variable has to be included in a prognostic regression model. 

Building MV might represent an alternative strategy to keep all the available information for the survival modelling analysis. Applying the PCAmix as data reduction method, the explainability of each MV is well achieved, as each original variable can contribute just for one of the five identified MV. Finally, the clinical significance of higher and lower values of each score is easily understandable by inspecting the correlation/associations plots, as shown in Supplementary Figures S2–S6.

Our results confirm the prognostic relevance of nodal involvement, summarized in NODAL^MV^, for all the outcomes analyzed. The highest squared loading for the NODAL^MV^ were observed for N category, the nodal ratio and number of positive nodes (Supplementary Table S10) underlining the relevant weight of these variables for the definition of the score and, therefore, their prognostic value. Such an observation is in keeping with the robust literature of recent decades [4,42], including the last update of the TNM classification system [43]. Features such as the nodal ratio [5] and the total number of positive nodes [44] are of main interest and were also tested for the proposal of a new staging systems in the OCSCC setting [45], which are easily available from any pathologic report.

One of the main findings obtained from this analysis suggests that tumor CD8^+^ T-cells depletion, derived by measuring CD8^+^ T-cell density in different tumoral compartments, and summarized within the IT-CD8^MV^, is associated with a higher risk of distant failure, independently from the NODAL^MV^ score. Interestingly, the correlation with DRFS apparently displays a threshold effect, with a critical IT-CD8^MV^ score below which the risk of distant metastasis steadily increases (Figure 6D). Although limited to few studies, an association between high tumor CD8^+^ T-cell density and a better distant metastasis free-survival was already observed in solid tumors from different primary sites including breast [46], colon [47] or soft tissues [48]. Among head and neck malignancies, evidence derived from the analysis of nasopharyngeal carcinoma [49], hypopharyngeal carcinoma [50,51] or from carcinomas of mixed primary sites [52] further support this finding. For OSCC, the association of CD8^+^ T-cell infiltration and distant failure still needs to be better defined [53]; however, indirect but still limited findings [52], support their protective role. 

The occurrence of metastatic disease is associated with systemic immune escape [54]. Cancer-cell intrinsic features as well as host response might represent relevant players in the T-cell exclusion (TCE) mechanism [55,56,57]. No data are available on the molecular basis sustaining TCE in OSCC. However, TCE can be bypassed by various immunotherapy strategies [58,59,60,61,62,63]. Currently, immune checkpoint inhibitors are investigated in the neoadjuvant setting in OCSCC, and this could allegedly enhance CD8^+^ T-cell activity and reverse tumor host interplay, thus reducing also the risk of tumor escape at distant sites [64,65,66,67,68].

Notably, as recently proposed, photodynamic therapy (PDT) combined with CTLA-4 blockade can enhance the cytotoxic CD8^+^ T-cell response to achieve durable tumor eradication and inducing an immunological memory [69]. This is worthwhile, since PDT is one of the treatment options for early or recurrent oral cavity squamous cell carcinoma [70,71] and its possible combination with immune checkpoint blockade drugs could pave the way for new trials design.

The main limits of our study are the retrospective design and the mono-institutional setting. Prospective and multi-institutional validation represents mandatory requirements to confirm our observations.

## 5. Conclusions

The results obtained from this study using data reduction methods confirm the key role of nodal involvement and intratumor CD8^+^ T-cell density on relevant survival endpoints. Further investigations for the identification of TCE mechanisms will help to identify appropriate treatment strategies for the subgroup of CD8^+^ T-cell in poor OSCC.

## Figures and Tables

**Table 1:** Summary statistics of the cohort.

Variable	Overall (N = 182)	Variable	Overall (N = 182)
**Age**		**Margins**	
Mean (SD)	63.6 (13.1)	Positive	32 (17.6%)
Median (Min, Max)	64.0 (26.0, 93.0)	Close	54 (29.7%)
**Sex**		Negative	96 (52.7%)
Male	115 (63.2%)	**Treatment**	
Female	67 (36.8%)	Surgery	78 (42.9%)
**pT category (8th Ed.)**		Surgery+RT	61 (33.5%)
pT1	23 (12.6%)	Surgery+CRT	43 (23.6%)
pT2	41 (22.5%)	**WBC (10^9^/L)**	
pT3	85 (46.7%)	Mean (SD)	7.41 (1.94)
pT4	33 (18.1%)	Median (Min, Max)	7.39 (2.77, 13.7)
**pN category (8th Ed.)**		**Lymphocytes (10^9^/L)**	
pN0	96 (52.7%)	Mean (SD)	1.82 (0.535)
pN1	24 (13.2%)	Median (Min, Max)	1.78 (0.690, 3.69)
pN2a	6 (3.3%)	**Neutrophils (10^9^/L)**	
pN2b	16 (8.8%)	Mean (SD)	4.79 (1.64)
pN2c	2 (1.1%)	Median (Min, Max)	4.76 (1.04, 10.1)
pN3b	38 (20.9%)	**Monocytes (10^9^/L)**	
**Npos**		Mean (SD)	0.585 (0.231)
pN0	97 (53.3%)	Median (Min, Max)	0.550 (0.160, 1.53)
pN+	85 (46.7%)	**PLT (10^9^/L)**	
**ENE**		Mean (SD)	228 (68.6)
No	140 (76.9%)	Median (Min, Max)	220 (51.0, 431)
Yes	42 (23.1%)	**NLR**	
**Nodal ratio**		Mean (SD)	2.83 (1.31)
Mean (SD)	0.0326 (0.0558)	Median (Min, Max)	2.53 (0.799, 7.55)
Median (Min, Max)	0 (0, 0.320)	**PLR**	
**Total Number Positive Nodes**		Mean (SD)	134 (52.7)
Mean (SD)	1.54 (2.84)	Median (Min, Max)	129 (31.1, 315)
Median (Min, Max)	0 (0, 18.0)	**CD8 density Total (cells/mm^2^)**	
**LVI**		Mean (SD)	418 (403)
No	130 (71.4%)	Median (Min, Max)	279 (16, 2180)
Yes	52 (28.6%)	**CD8 density CT (cells/mm^2^)**	
**PNI**		Mean (SD)	333 (406)
No	92 (50.5%)	Median (Min, Max)	170 (5, 2270)
Yes	90 (49.5%)	**CD8 density IM (cells/mm^2^)**	
**Differentiation**		Mean (SD)	520 (429)
G1	18 (9.9%)	Median (Min, Max)	394 (24, 2340)
G2	87 (47.8%)		
G3	77 (42.3%)		
**Bone invasion**			
No	148 (81.3%)		
Cortical	16 (8.8%)		
Medullary	18 (9.9%)		

## Data Availability

The full dataset will be available upon reasonable request to the corresponding author.

## Supplementary Materials

The following are available online at https://www.mdpi.com/article/10.3390/cells10092203/s1, Supplementary Figure S1: Heatmap showing the missing data in the whole cohort (A); intersection plot of missing data (B). Supplementary Figure S2: Scatter plots showing the relationship between TI-CD8^MV^ and the variables from which it is defined. P values are estimated by Spearman correlation test. Supplementary Figure S3: Box plots and scatter plots showing the relationship between the NODAL^MV^ and the variables from which it is defined. P values are estimated by Kruskal–Wallis test, Wilcoxon sign-rank test or Spearman correlation test. Supplementary Figure S4: Scatter plots and box plots showing the relationship between the MYELOID^MV^ and the variables from which it is defined. P values are estimated by the Spearman correlation test or Wilcoxon sign-rank test. Supplementary Figure S5: Scatter plots showing the relationship between the LYMPHOID^MV^ and the variables from which it is defined. P values are estimated by the Spearman correlation test. Supplementary Figure S6: Box plots showing the relationship between the TUMOR^MV^ and the variables from which it is defined. P values are estimated by Kruskal–Wallis test. Supplementary Table S1: R values of Spearman’s correlation analysis between continuous variables analyzed. Legend: ****, *p* < 0.0001; ***, *p* < 0.001; **, *p* < 0.01; *, *p* < 0.05. Supplementary Table S2: Association analysis between peripheral or intratumoral biomarkers measures and sex. P values estimated by Wilcoxon test. Supplementary Table S3: Association analysis between peripheral or intratumoral biomarkers measures and pT category. P values estimated by Kruskal–Wallis test. Supplementary Table S4: Association analysis between peripheral or intratumoral biomarkers measures and pN category. P values estimated by Kruskal–Wallis test. Supplementary Table S5: Association analysis between peripheral or intratumoral biomarkers measures and extranodal extension (ENE). P values estimated by Wilcoxon test. Supplementary Table S6: Association analysis between peripheral or intratumoral biomarkers measures and grading. P values estimated by Kruskal–Wallis test. Supplementary Table S7: Association analysis between peripheral or intratumoral biomarkers measures and bone invasion. P values estimated by Kruskal–Wallis test. Supplementary Table S8: Association analysis between peripheral or intratumoral biomarkers measures and perineural invasion (PNI). P values estimated by Wilcoxon test. Supplementary Table S9: Association analysis between peripheral or intratumoral biomarkers measures and lymphovascular invasion (LVI). P values estimated by Wilcoxon test. Supplementary Table S10: Details of the loadings for each variable and level composing the 5 metavariables (^MV^). Supplementary Table S11: Extensive details of multivariable survival Cox proportional hazards models. Legend: d.f., degrees of freedom; Coefficient, regression coefficient; S.E., standard error.
