# Supplementary material for: Metavariables Resuming Host Immune Features and Nodal Involvement Are Associated with Oncological Outcomes in Oral Cavity Squamous Cell Carcinoma
Source: Cells. 2021 Aug 26;10(9):2203. doi: 10.3390/cells10092203 (PMC8472482; doi:10.3390/cells10092203)
Supplement: Supplementary file 1 [file cells-10-02203-s001.zip › cells-1324845-supplementary.pdf]

cells

MDPI

Metavariables Resuming Host Immune Features and Nodal Involvement Are Associated with Oncological Outcomes in Oral Cavity Squamous Cell Carcinoma

Francesco Missale,[1,2*] Mattia Bugatti,[3] Davide Mattavelli,[4] Silvia Lonardi,[3] Davide Lombardi,[4] Piero Nicolai,[5] Cesare Piazza,[4] Simonetta Battocchio,[3] Anna Bozzola,[3] Stefano Calza[6,7,8] and William Vermi[1,3,9*]

[1] Department of Molecular and Translational Medicine, University of Brescia, 25125 Brescia, Italy

[2] Department of Head & Neck Oncology & Surgery Otorhinolaryngology, Antoni Van Leeuwenhoek, Nederlands Kanker Instituut, 1066 Amsterdam, The Netherlands

[3] Unit of Pathology, ASST Spedali Civili di Brescia, 25100 Brescia, Italy

[4] Unit of Otorhinolaryngology—Head and Neck Surgery, Department of Medical and Surgical Specialties, Radiological Sciences, and Public Health, University of Brescia, 25123 Brescia, Italy

[5] Section of Otorhinolaryngology—Head and Neck Surgery, Department of Neurosciences, University of Padua, Via Giustiniani, 2-35128 Padua, Italy;

[6] Unit of Biostatistics, Department of Molecular and Translational Medicine, University of Brescia, 25125 Brescia, Italy

[7] Department of Medical Epidemiology and Biostatistics, Karolinska Institutet, 17177 Stockholm, Sweden

[8] Big & Open Data Innovation Laboratory, University of Brescia, 25125 Brescia, Italy

[9] Department of Pathology and Immunology, Washington University School of Medicine, St. Louis, MO 63130, USA

***Correspondence to: Francesco Missale and William Vermi**

Department of Molecular and Translational Medicine, University of Brescia,

Viale Europa, 11; 25123, Brescia, Italy

Email: f.missale@unibs.it; william.vermi@unibs.it

cells

MDPI

Supplementary Material

Supplementary Figures

Supplementary Figure S1: Heatmap showing the missingness data in the whole cohort (**A**); intersection plot of missing data (**B**).

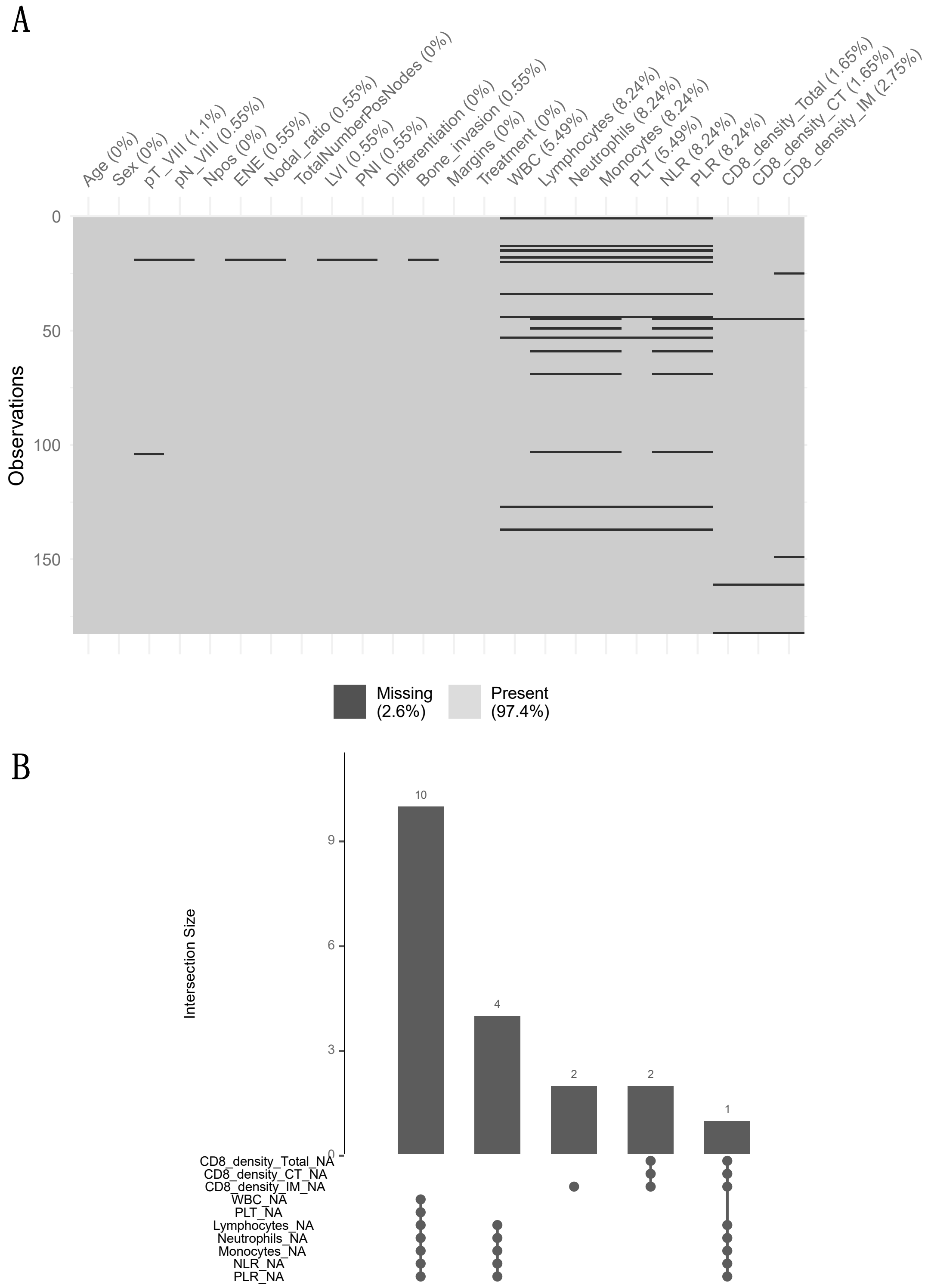

Supplementary Figure S2: Scatter plots showing the relationship between TI-CD8^{MV} and the variables from which it is defined. P values are estimated by Spearman correlation test.

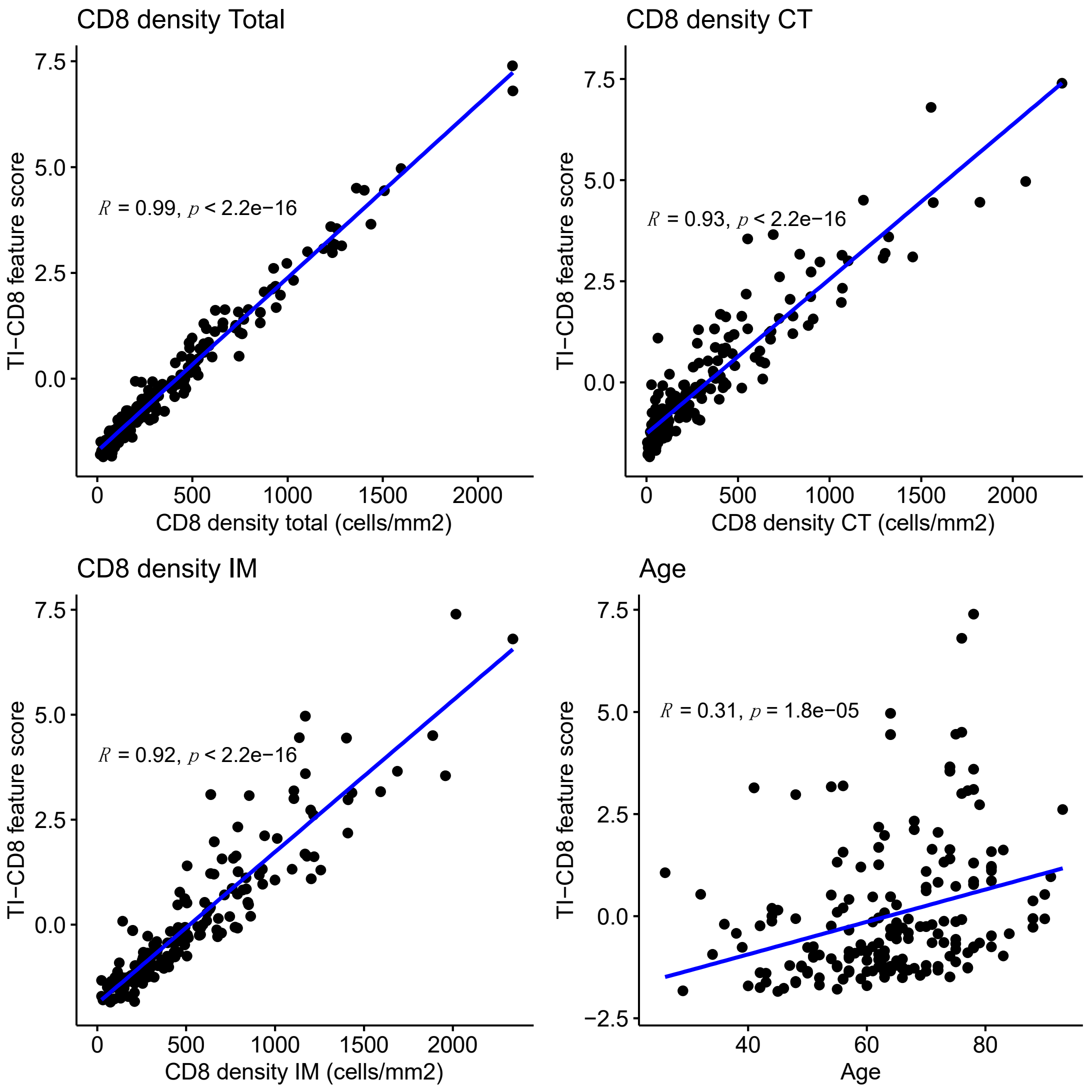

Supplementary Figure S3: Box plots and scatter plots showing the relationship between the NODAL^{MV} and the variables from which it is defined. P values are estimated by Kruskal-Wallis test, Wilcoxon sign-rank test or Spearman correlation test.

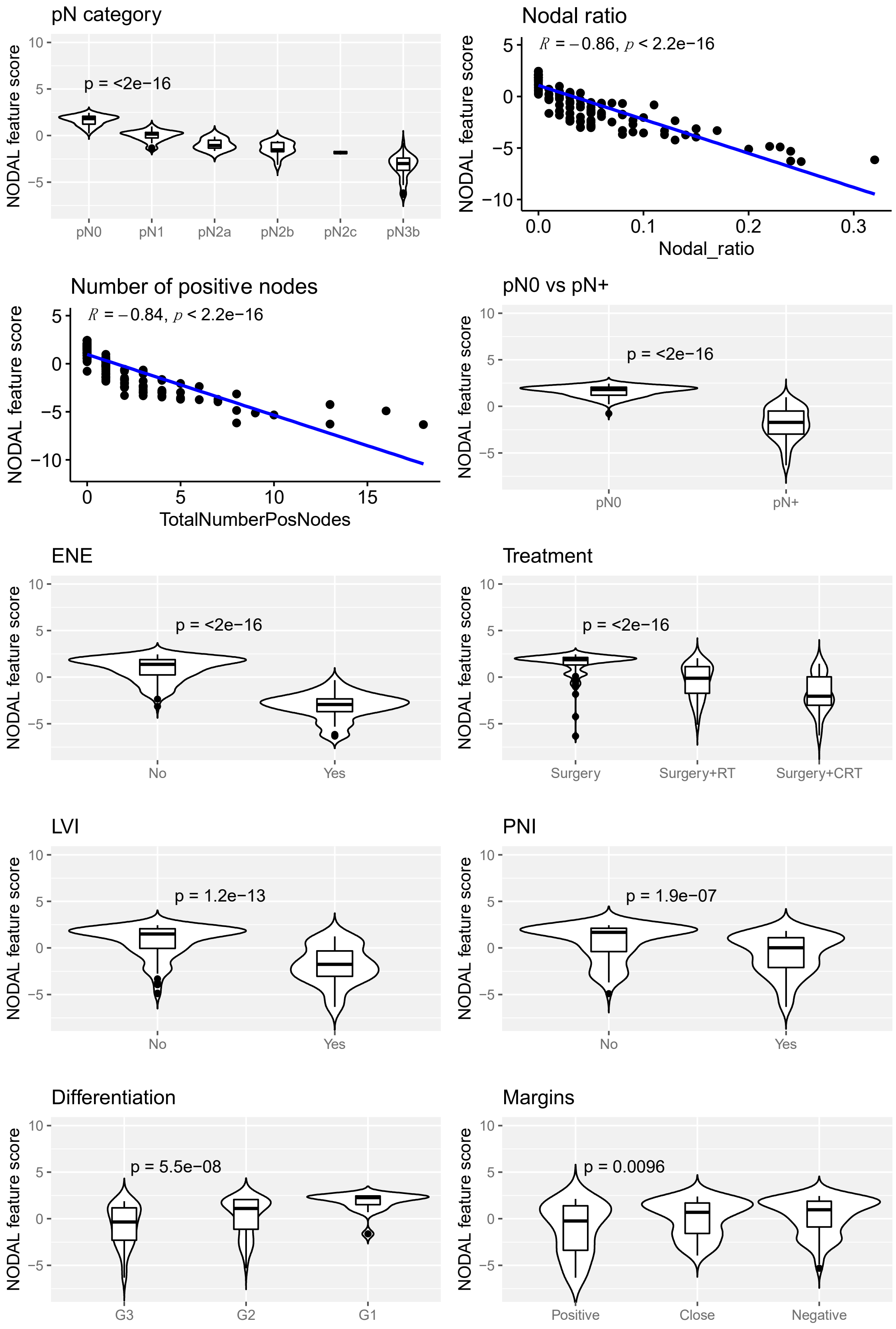

Supplementary Figure S4: Scatter plots and box plots showing the relationship between the MYELOID^{MV} and the variables from which it is defined. P values are estimated by theSpearman correlation test or Wilcoxon sign-rank test.

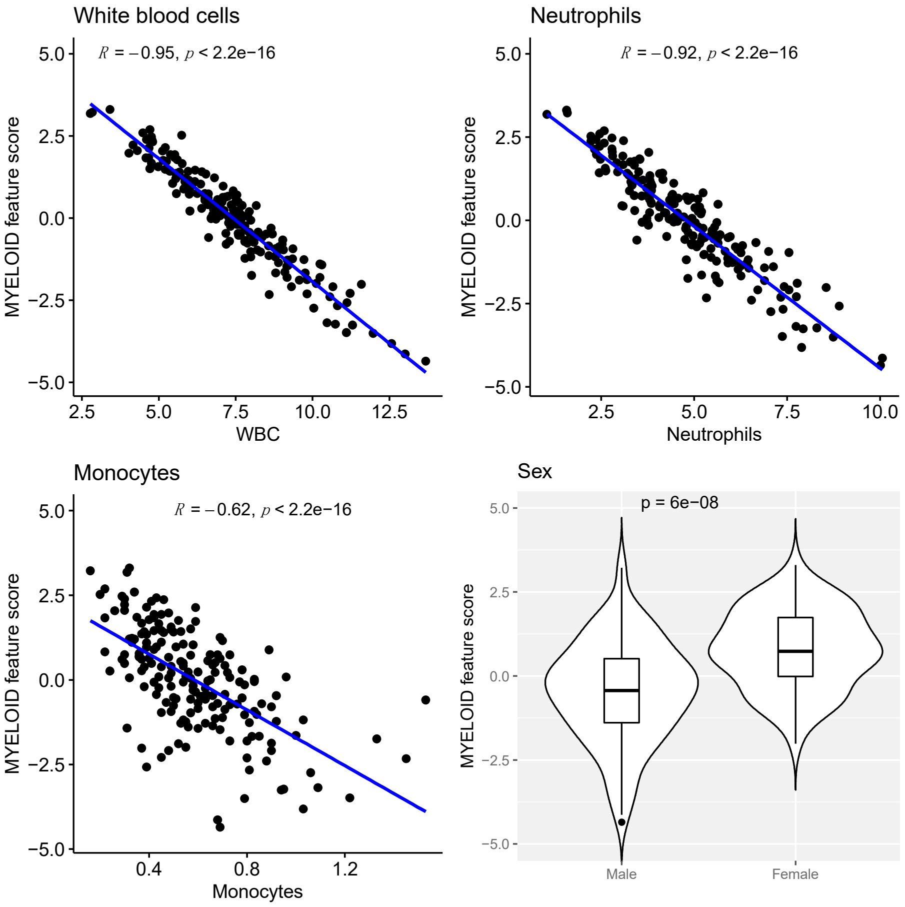

cells

Supplementary Figure S5: Scatter plots showing the relationship between the LYMPHOID[MV] and the variables from which it is defined. P values are estimated by Spearman correlation test.

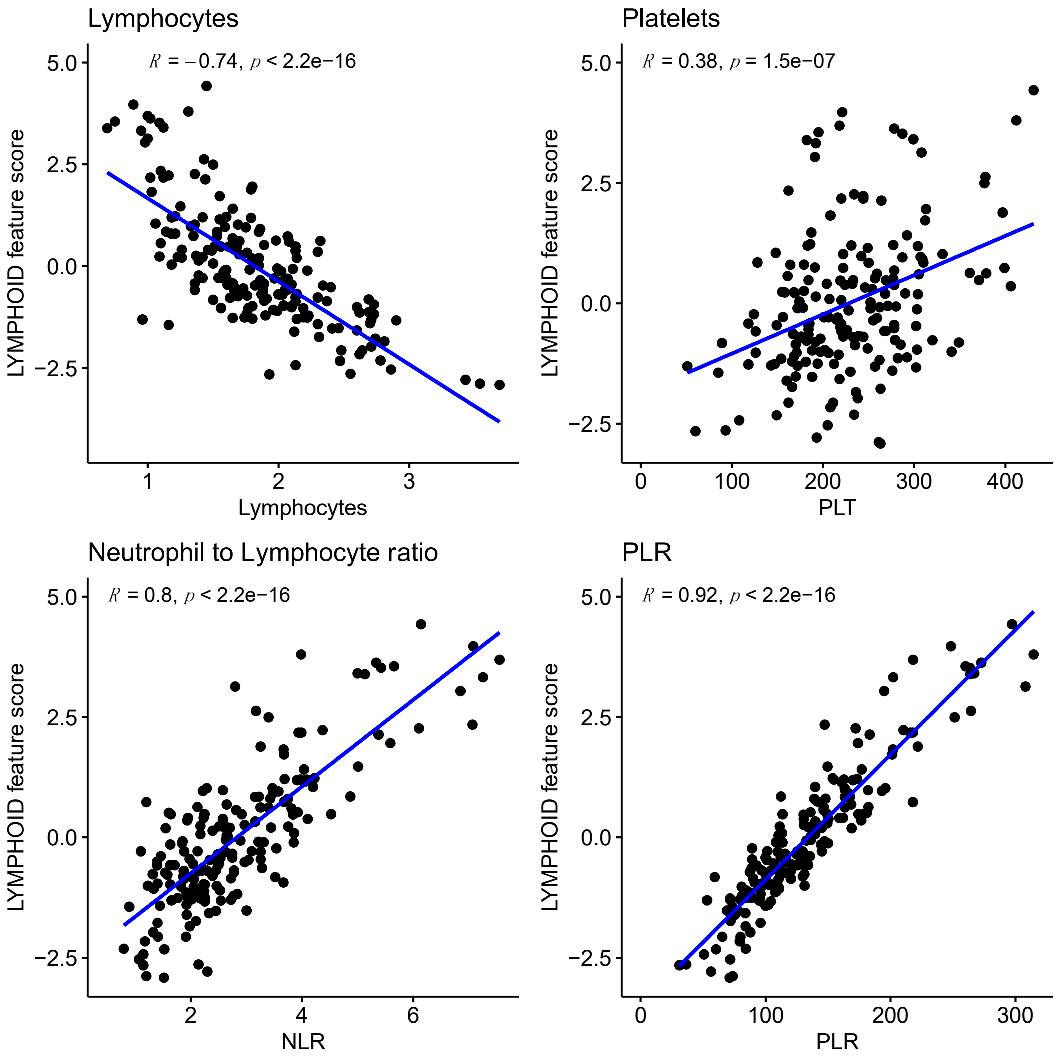

cells

Supplementary Figure S6: Box plots showing the relationship between the $TUMOR^{MV}$ and the variables from which it is defined. P values are estimated by Kruskal-Wallis test.

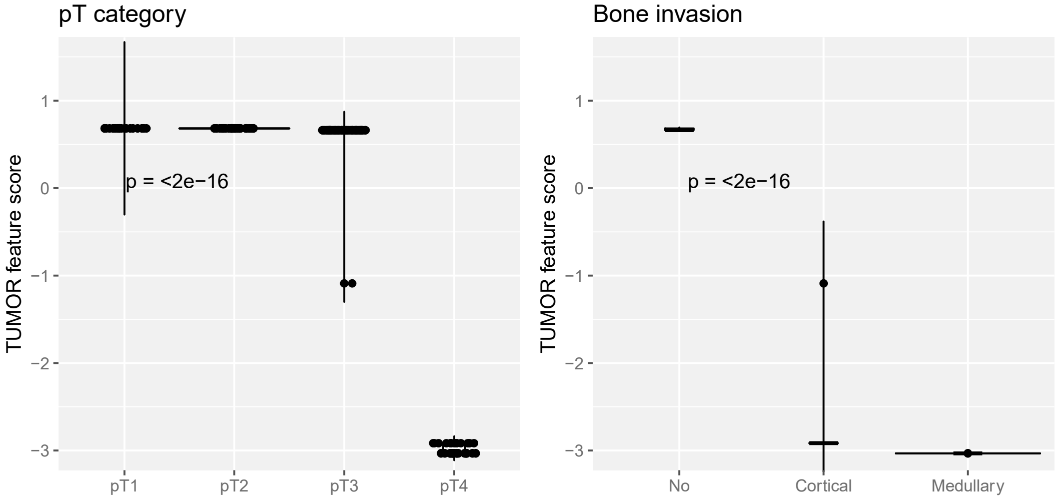

Supplementary Tables

Supplementary Table S1: R values of the Spearman's correlation analysis between continuous variables analyzed. Legend: ****, $p < 0.0001$; ***, $p < 0.001$; **, $p < 0.01$; *, $p < 0.05$.

	Age	Nodal Ratio	N° Positive Nodes	WBC	Lymphocytes	Neutrophils	Monocytes	PLT	NLR	PLR	^{TOT}CD8 density	^{CT}CD8 density
**Age**												
**Nodal Ratio**	-0.03											
**N° Positive Nodes**	-0.04	0.98****										
**WBC**	-0.19*	-0.01	0.01									
**Lymphocytes**	-0.16*	-0.04	-0.01	0.41****								
**Neutrophils**	-0.17*	0.00	0.02	0.94****	0.15*							
**Monocytes**	0.02	0.01	0.05	0.51****	0.20**	0.37****						
**PLT**	-0.09	-0.06	-0.04	0.30****	0.26***	0.26***	0.16*					
**NLR**	-0.03	0.03	0.03	0.48****	-0.52****	0.71****	0.16*	0.03				
**PLR**	0.05	-0.04	-0.05	-0.07	-0.57****	0.12	-0.03	0.57****	0.49****			
**^{TOT}CD8 density**	0.22**	0.11	0.11	-0.02	0.06	-0.04	-0.10	0.02	-0.07	-0.04		
**^{CT}CD8 density**	0.21**	0.20**	0.20**	-0.02	0.05	-0.04	-0.11	0.03	-0.04	-0.03	0.92****	
**^{IM}CD8 density**	0.24**	0.01	0.01	-0.06	-0.01	-0.08	-0.10	-0.04	-0.06	-0.02	0.92****	0.78****

cells

MDPI

Supplementary Table S2: Association analysis between peripheral or intratumoral biomarkers measures and Sex. P values estimated by Wilcoxon test.

	Male (N=115)	Female (N=67)	P-value
**WBC (10^9/L)**			
Mean (SD)	7.75 (2.05)	6.84 (1.60)	0.002
Median [Min, Max]	7.65 [2.77, 13.7]	6.91 [3.41, 11.6]	
**Lymphocytes (10^9/L)**			
Mean (SD)	1.82 (0.587)	1.81 (0.438)	0.881
Median [Min, Max]	1.80 [0.690, 3.69]	1.76 [1.00, 2.78]	
**Neutrophils (10^9/L)**			
Mean (SD)	5.07 (1.70)	4.31 (1.42)	0.002
Median [Min, Max]	4.97 [1.04, 10.1]	4.15 [1.57, 8.55]	
**Monocytes (10^9/L)**			
Mean (SD)	0.613 (0.234)	0.535 (0.219)	0.018
Median [Min, Max]	0.580 [0.160, 1.45]	0.490 [0.200, 1.53]	
**PLT (10^9/L)**			
Mean (SD)	219 (68.0)	243 (67.4)	0.015
Median [Min, Max]	215 [51.0, 431]	242 [60.0, 412]	
**NLR (10^9/L)**			
Mean (SD)	3.02 (1.40)	2.50 (1.07)	0.015
Median [Min, Max]	2.65 [0.897, 7.25]	2.26 [0.799, 7.55]	
**PLR (10^9/L)**			
Mean (SD)	130 (56.1)	140 (46.0)	0.057
Median [Min, Max]	113 [36.5, 308]	132 [31.1, 315]	
**^{TOT}CD8 density (/mm^2)**			
Mean (SD)	385 (352)	473 (477)	0.237
Median [Min, Max]	244 [16.4, 1440]	290 [42.2, 2180]	
**^{CT}CD8 density (/mm^2)**			
Mean (SD)	297 (338)	395 (500)	0.316
Median [Min, Max]	132 [5.13, 1820]	213 [9.96, 2270]	
**^{IM}CD8 density (/mm^2)**			
Mean (SD)	500 (417)	554 (448)	0.288
Median [Min, Max]	361 [23.6, 1960]	437 [79.6, 2340]	

cells

MDPI

Supplementary Table S3: Association analysis between peripheral or intratumoral biomarkers measures and pT category. P values estimated by Kruskal-Wallis test.

	pT1 (N=23)	pT2 (N=41)	pT3 (N=85)	pT4 (N=33)	P-value
**WBC (10^9/L)**					
Mean (SD)	7.83 (2.52)	7.11 (1.54)	7.46 (1.98)	7.38 (1.85)	0.701
Median [Min, Max]	7.59 [3.41, 12.6]	6.99 [4.30, 11.1]	7.47 [2.77, 13.7]	7.17 [4.67, 13.0]	
**Lymphocytes (10^9/L)**					
Mean (SD)	2.01 (0.681)	1.73 (0.480)	1.83 (0.562)	1.76 (0.382)	0.245
Median [Min, Max]	2.02 [0.690, 3.43]	1.65 [0.980, 2.70]	1.79 [0.750, 3.69]	1.69 [1.03, 2.71]	
**Neutrophils (10^9/L)**					
Mean (SD)	5.06 (1.98)	4.55 (1.29)	4.82 (1.69)	4.83 (1.70)	0.745
Median [Min, Max]	5.11 [1.57, 8.74]	4.75 [2.22, 7.77]	4.72 [1.04, 10.0]	4.46 [2.48, 10.1]	
**Monocytes (10^9/L)**					
Mean (SD)	0.579 (0.200)	0.603 (0.272)	0.584 (0.209)	0.568 (0.257)	0.88
Median [Min, Max]	0.510 [0.220, 1.03]	0.520 [0.290, 1.45]	0.590 [0.160, 1.33]	0.540 [0.220, 1.53]	
**PLT (10^9/L)**					
Mean (SD)	230 (56.7)	224 (68.7)	220 (62.9)	253 (84.9)	0.243
Median [Min, Max]	217 [108, 349]	208 [60.0, 399]	220 [51.0, 431]	233 [89.0, 412]	
**NLR (10^9/L)**					
Mean (SD)	2.77 (1.51)	2.88 (1.38)	2.83 (1.31)	2.81 (1.10)	0.927
Median [Min, Max]	2.34 [1.11, 7.08]	2.51 [1.15, 7.06]	2.59 [0.799, 7.55]	2.65 [1.23, 5.59]	
**PLR (10^9/L)**					
Mean (SD)	130 (61.2)	139 (54.5)	128 (49.2)	145 (52.5)	0.181
Median [Min, Max]	116 [50.7, 264]	130 [31.1, 308]	120 [36.5, 297]	138 [59.3, 315]	
**^{TOT}CD8 density (/mm^2)**					
Mean (SD)	341 (353)	496 (449)	411 (383)	392 (427)	0.315
Median [Min, Max]	207 [61.8, 1250]	339 [22.2, 2180]	281 [16.4, 1600]	290 [31.0, 2180]	
**^{CT}CD8 density (/mm^2)**					
Mean (SD)	199 (275)	367 (410)	347 (419)	349 (443)	0.104
Median [Min, Max]	72.2 [9.09, 947]	215 [21.9, 1570]	173 [5.13, 2070]	208 [17.5, 2270]	
**^{IM}CD8 density (/mm^2)**					
Mean (SD)	442 (419)	637 (470)	494 (386)	496 (477)	0.117
Median [Min, Max]	288 [96.9, 1590]	489 [23.6, 2340]	396 [23.6, 1960]	393 [56.3, 2020]	

cells MDPI

Supplementary Table S4: Association analysis between peripheral or intratumoral biomarkers measures and pN category. P values estimated by Kruskal-Wallis test.

	pN0 (N=96)	pN1 (N=24)	pN2a (N=6)	pN2b (N=16)	pN2c (N=2)	pN3b (N=38)	P-value
**WBC (10^9/L)**							
Mean (SD)	7.56 (2.02)	6.88 (1.77)	6.30 (1.82)	6.70 (1.60)	9.70 (2.27)	7.73 (1.83)	0.099
Median [Min, Max]	7.39 [3.41, 13.0]	6.90 [2.77, 10.7]	6.78 [2.84, 7.88]	7.30 [4.16, 9.82]	9.70 [8.09, 11.3]	7.80 [4.03, 13.7]	
**Lymphocytes (10^9/L)**							
Mean (SD)	1.84 (0.556)	1.85 (0.402)	1.54 (0.667)	1.60 (0.464)	2.06 (0.0283)	1.87 (0.566)	0.299
Median [Min, Max]	1.79 [0.750, 3.54]	1.88 [1.12, 2.70]	1.50 [0.890, 2.74]	1.61 [0.690, 2.37]	2.06 [2.04, 2.08]	1.79 [1.02, 3.69]	
**Neutrophils (10^9/L)**							
Mean (SD)	4.91 (1.74)	4.23 (1.40)	4.07 (1.54)	4.37 (1.28)	6.44 (2.11)	5.05 (1.57)	0.173
Median [Min, Max]	4.81 [1.57, 10.1]	4.29 [1.04, 7.55]	4.20 [1.59, 6.30]	4.59 [2.22, 7.33]	6.44 [4.94, 7.93]	5.22 [2.32, 10.0]	
**Monocytes (10^9/L)**							
Mean (SD)	0.590 (0.263)	0.606 (0.204)	0.532 (0.193)	0.483 (0.193)	0.835 (0.148)	0.595 (0.165)	0.14
Median [Min, Max]	0.535 [0.200, 1.53]	0.610 [0.220, 1.03]	0.605 [0.160, 0.670]	0.450 [0.220, 0.920]	0.835 [0.730, 0.940]	0.580 [0.300, 1.06]	
**PLT (10^9/L)**							
Mean (SD)	231 (66.2)	227 (72.7)	211 (114)	232 (92.2)	304 (1.41)	218 (52.5)	0.53
Median [Min, Max]	222 [89.0, 431]	219 [85.0, 379]	227 [51.0, 378]	198 [60.0, 412]	304 [303, 305]	220 [118, 312]	
**NLR (10^9/L)**							
Mean (SD)	2.90 (1.41)	2.28 (0.715)	3.07 (2.08)	2.94 (1.11)	2.61 (1.70)	2.94 (1.23)	0.391
Median [Min, Max]	2.53 [0.799, 7.55]	2.19 [0.897, 3.94]	2.55 [1.41, 7.08]	2.73 [1.15, 5.13]	2.61 [1.41, 3.81]	2.67 [1.07, 7.06]	
**PLR (10^9/L)**							
Mean (SD)	134 (48.6)	125 (37.6)	147 (90.7)	159 (79.0)	122 (35.4)	126 (50.2)	0.621
Median [Min, Max]	130 [50.7, 297]	126 [36.5, 218]	123 [53.1, 264]	148 [31.1, 315]	122 [96.6, 147]	112 [65.3, 273]	
**^{TOT}CD8 density (/mm^2)**							
Mean (SD)	393 (400)	481 (506)	375 (374)	507 (424)	383 (102)	412 (353)	0.881
Median [Min, Max]	253 [16.4, 2180]	289 [71.7, 2180]	262 [70.1, 1100]	374 [49.7, 1360]	383 [310, 455]	331 [22.2, 1440]	
**^{CT}CD8 density (/mm^2)**							
Mean (SD)	291 (394)	406 (539)	314 (402)	428 (402)	211 (72.9)	363 (358)	0.335
Median [Min, Max]	120 [5.13, 2070]	149 [27.7, 2270]	162 [40.0, 1100]	329 [20.7, 1300]	211 [159, 262]	289 [18.3, 1820]	
**^{IM}CD8 density (/mm^2)**							
Mean (SD)	525 (433)	511 (460)	467 (342)	645 (506)	434 (52.2)	473 (396)	0.904
Median [Min, Max]	350 [31.9, 2340]	350 [99.0, 2020]	407 [122, 1110]	482 [56.3, 1890]	434 [397, 471]	346 [23.6, 1690]	

Supplementary Table S5: Association analysis between peripheral or intratumoral biomarkers measures and Extranodal extension (ENE). P values estimated by Wilcoxon test.

	ENE No (N=140)	ENE Yes (N=42)	P-value
**WBC (10^9/L)**			
Mean (SD)	7.35 (1.97)	7.62 (1.86)	0.221
Median [Min, Max]	7.27 [2.77, 13.0]	7.69 [2.84, 13.7]	
**Lymphocytes (10^9/L)**			
Mean (SD)	1.81 (0.519)	1.84 (0.592)	0.879
Median [Min, Max]	1.79 [0.690, 3.54]	1.75 [0.890, 3.69]	
**Neutrophils (10^9/L)**			
Mean (SD)	4.73 (1.67)	4.99 (1.57)	0.181
Median [Min, Max]	4.46 [1.04, 10.1]	5.10 [1.59, 10.0]	
**Monocytes (10^9/L)**			
Mean (SD)	0.583 (0.247)	0.589 (0.170)	0.369
Median [Min, Max]	0.540 [0.200, 1.53]	0.580 [0.160, 1.06]	
**PLT (10^9/L)**			
Mean (SD)	233 (70.9)	212 (58.0)	0.178
Median [Min, Max]	221 [60.0, 431]	220 [51.0, 312]	
**NLR (10^9/L)**			
Mean (SD)	2.78 (1.29)	2.98 (1.36)	0.345
Median [Min, Max]	2.51 [0.799, 7.55]	2.67 [1.07, 7.08]	
**PLR (10^9/L)**			
Mean (SD)	137 (52.4)	125 (53.0)	0.076
Median [Min, Max]	131 [31.1, 315]	111 [53.1, 273]	
**^{TOT}CD8 density (/mm^2)**			
Mean (SD)	424 (421)	397 (340)	0.869
Median [Min, Max]	279 [16.4, 2180]	295 [22.2, 1440]	
**^{CT}CD8 density (/mm^2)**			
Mean (SD)	329 (424)	345 (347)	0.225
Median [Min, Max]	156 [5.13, 2270]	271 [18.3, 1820]	
**^{IM}CD8 density (/mm^2)**			
Mean (SD)	536 (442)	465 (379)	0.391
Median [Min, Max]	384 [31.9, 2340]	403 [23.6, 1690]	

cells

MDPI

Supplementary Table S6: Association analysis between peripheral or intratumoral biomarkers measures and grading. P values estimated by Kruskal-Wallis test.

	G3 (N=77)	G2 (N=87)	G1 (N=18)	P-value
**WBC (10^9/L)**				
Mean (SD)	7.54 (2.09)	7.34 (1.84)	7.23 (1.84)	0.738
Median [Min, Max]	7.58 [2.77, 13.7]	7.19 [4.03, 12.0]	7.27 [3.41, 11.1]	
**Lymphocytes (10^9/L)**				
Mean (SD)	1.82 (0.556)	1.81 (0.543)	1.85 (0.423)	0.901
Median [Min, Max]	1.80 [0.750, 3.69]	1.72 [0.690, 3.54]	1.83 [1.23, 2.71]	
**Neutrophils (10^9/L)**				
Mean (SD)	4.90 (1.74)	4.74 (1.55)	4.55 (1.74)	0.557
Median [Min, Max]	4.92 [1.04, 10.1]	4.46 [2.22, 8.74]	4.09 [1.57, 8.90]	
**Monocytes (10^9/L)**				
Mean (SD)	0.578 (0.208)	0.577 (0.229)	0.651 (0.322)	0.779
Median [Min, Max]	0.570 [0.160, 1.03]	0.540 [0.200, 1.45]	0.530 [0.320, 1.53]	
**PLT (10^9/L)**				
Mean (SD)	231 (73.6)	223 (62.0)	241 (77.5)	0.481
Median [Min, Max]	233 [51.0, 397]	218 [89.0, 412]	220 [126, 431]	
**NLR (10^9/L)**				
Mean (SD)	2.91 (1.37)	2.82 (1.23)	2.53 (1.40)	0.3
Median [Min, Max]	2.54 [0.897, 7.08]	2.60 [0.799, 7.55]	2.14 [1.11, 6.14]	
**PLR (10^9/L)**				
Mean (SD)	136 (57.1)	132 (47.3)	135 (59.7)	0.966
Median [Min, Max]	126 [31.1, 308]	130 [36.5, 315]	123 [60.3, 297]	
**^{TOT}CD8 density (/mm^2)**				
Mean (SD)	404 (370)	435 (456)	395 (249)	0.613
Median [Min, Max]	294 [18.6, 2180]	231 [16.4, 2180]	351 [42.2, 996]	
**^{CT}CD8 density (/mm^2)**				
Mean (SD)	322 (392)	358 (446)	261 (243)	0.917
Median [Min, Max]	191 [5.13, 2270]	152 [8.38, 2070]	235 [22.0, 899]	
**^{IM}CD8 density (/mm^2)**				
Mean (SD)	516 (402)	513 (463)	573 (379)	0.477
Median [Min, Max]	420 [23.6, 2020]	305 [23.6, 2340]	481 [95.8, 1260]	

cells

MDPI

Supplementary Table S7: Association analysis between peripheral or intratumoral biomarkers measures and bone invasion. P values estimated by Kruskal-Wallis test.

	No bone invasion (N=148)	Cortical invasion (N=16)	Medullary invasion (N=18)	P-value
**WBC (10^9/L)**				
Mean (SD)	7.41 (1.97)	7.08 (2.13)	7.76 (1.56)	0.378
Median [Min, Max]	7.43 [2.77, 13.7]	6.51 [4.67, 13.0]	7.62 [4.71, 10.7]	
**Lymphocytes (10^9/L)**				
Mean (SD)	1.83 (0.562)	1.81 (0.492)	1.76 (0.316)	0.92
Median [Min, Max]	1.79 [0.690, 3.69]	1.71 [1.03, 2.71]	1.72 [1.31, 2.32]	
**Neutrophils (10^9/L)**				
Mean (SD)	4.77 (1.64)	4.55 (1.89)	5.15 (1.48)	0.341
Median [Min, Max]	4.78 [1.04, 10.0]	3.81 [2.48, 10.1]	5.16 [2.58, 7.74]	
**Monocytes (10^9/L)**				
Mean (SD)	0.589 (0.226)	0.544 (0.309)	0.588 (0.196)	0.41
Median [Min, Max]	0.565 [0.160, 1.45]	0.520 [0.240, 1.53]	0.550 [0.220, 1.09]	
**PLT (10^9/L)**				
Mean (SD)	223 (63.6)	228 (67.7)	273 (92.7)	0.056
Median [Min, Max]	220 [51.0, 431]	219 [126, 341]	278 [89.0, 412]	
**NLR (10^9/L)**				
Mean (SD)	2.84 (1.36)	2.57 (1.11)	3.00 (1.05)	0.46
Median [Min, Max]	2.53 [0.799, 7.55]	2.31 [1.23, 5.59]	3.15 [1.32, 5.38]	
**PLR (10^9/L)**				
Mean (SD)	132 (52.6)	128 (42.1)	157 (58.5)	0.084
Median [Min, Max]	125 [31.1, 308]	118 [80.8, 202]	156 [59.3, 315]	
**^{TOT}CD8 density (/mm^2)**				
Mean (SD)	425 (399)	271 (224)	485 (529)	0.344
Median [Min, Max]	279 [16.4, 2180]	170 [31.0, 724]	396 [42.2, 2180]	
**^{CT}CD8 density (/mm^2)**				
Mean (SD)	331 (400)	224 (234)	445 (548)	0.56
Median [Min, Max]	170 [5.13, 2070]	108 [17.5, 799]	344 [22.0, 2270]	
**^{IM}CD8 density (/mm^2)**				
Mean (SD)	527 (420)	416 (299)	555 (585)	0.613
Median [Min, Max]	396 [23.6, 2340]	340 [59.8, 912]	402 [56.3, 2020]	

Supplementary Table S8: Association analysis between peripheral or intratumoral biomarkers measures and perineural invasion (PNI). P values estimated by Wilcoxon test.

	No (N=92)	Yes (N=90)	P-value
**WBC (10^9/L)**			
Mean (SD)	7.23 (1.98)	7.60 (1.89)	0.277
Median [Min, Max]	7.22 [2.77, 12.6]	7.50 [4.16, 13.7]	
**Lymphocytes (10^9/L)**			
Mean (SD)	1.81 (0.541)	1.82 (0.533)	0.831
Median [Min, Max]	1.79 [0.690, 3.43]	1.76 [0.890, 3.69]	
**Neutrophils (10^9/L)**			
Mean (SD)	4.61 (1.62)	4.98 (1.66)	0.208
Median [Min, Max]	4.46 [1.04, 8.74]	4.84 [2.22, 10.1]	
**Monocytes (10^9/L)**			
Mean (SD)	0.589 (0.251)	0.580 (0.210)	0.891
Median [Min, Max]	0.545 [0.160, 1.53]	0.560 [0.220, 1.33]	
**PLT (10^9/L)**			
Mean (SD)	226 (68.2)	230 (69.2)	0.586
Median [Min, Max]	218 [51.0, 399]	231 [60.0, 431]	
**NLR (10^9/L)**			
Mean (SD)	2.72 (1.26)	2.94 (1.35)	0.26
Median [Min, Max]	2.48 [0.799, 7.25]	2.62 [1.07, 7.55]	
**PLR (10^9/L)**			
Mean (SD)	133 (53.1)	134 (52.5)	0.698
Median [Min, Max]	127 [50.7, 308]	130 [31.1, 315]	
**^{TOT}CD8 density (/mm^2)**			
Mean (SD)	405 (396)	430 (412)	0.586
Median [Min, Max]	250 [18.6, 2180]	300 [16.4, 2180]	
**^{CT}CD8 density (/mm^2)**			
Mean (SD)	297 (344)	370 (461)	0.185
Median [Min, Max]	127 [5.13, 1550]	193 [8.38, 2270]	
**^{IM}CD8 density (/mm^2)**			
Mean (SD)	531 (448)	508 (410)	0.958
Median [Min, Max]	398 [23.6, 2340]	377 [23.6, 2020]	

Supplementary Table S9: Association analysis between peripheral or intratumoral biomarkers measures and lymphovascular invasion (LVI). P values estimated by Wilcoxon test.

	LVI No (N=130)	LVI Yes (N=52)	P-value
**WBC (10^9/L)**			
Mean (SD)	7.44 (2.00)	7.34 (1.79)	0.945
Median [Min, Max]	7.31 [2.77, 13.0]	7.58 [4.03, 13.7]	
**Lymphocytes (10^9/L)**			
Mean (SD)	1.84 (0.555)	1.75 (0.483)	0.407
Median [Min, Max]	1.80 [0.690, 3.69]	1.76 [1.00, 2.86]	
**Neutrophils (10^9/L)**			
Mean (SD)	4.77 (1.68)	4.83 (1.57)	0.704
Median [Min, Max]	4.58 [1.04, 10.1]	4.94 [2.32, 10.0]	
**Monocytes (10^9/L)**			
Mean (SD)	0.597 (0.248)	0.554 (0.181)	0.476
Median [Min, Max]	0.555 [0.160, 1.53]	0.545 [0.220, 1.06]	
**PLT (10^9/L)**			
Mean (SD)	228 (72.0)	229 (59.7)	0.765
Median [Min, Max]	220 [51.0, 431]	221 [118, 406]	
**NLR (10^9/L)**			
Mean (SD)	2.79 (1.33)	2.93 (1.25)	0.3
Median [Min, Max]	2.48 [0.799, 7.25]	2.66 [1.07, 7.55]	
**PLR (10^9/L)**			
Mean (SD)	132 (52.1)	139 (54.3)	0.5
Median [Min, Max]	125 [31.1, 315]	132 [60.3, 308]	
**^{TOT}CD8 density (/mm^2)**			
Mean (SD)	453 (433)	330 (301)	0.104
Median [Min, Max]	298 [18.6, 2180]	237 [16.4, 1440]	
**^{CT}CD8 density (/mm^2)**			
Mean (SD)	363 (446)	259 (273)	0.523
Median [Min, Max]	176 [5.13, 2270]	164 [8.38, 1450]	
**^{IM}CD8 density (/mm^2)**			
Mean (SD)	568 (460)	399 (308)	0.032
Median [Min, Max]	438 [23.6, 2340]	317 [31.9, 1690]	

Supplementary Table S10: Details of the loadings for each variable and level composing the 5 metavariables (^{MV}).

**IT-CD8^{MV}**	**squared loading**
CD8 density Total	0.973
CD8 density CT	0.862
CD8 density IM	0.853
Age	0.097
**NODAL^{MV}**	**squared loading**
pN VIII	0.870
Nodal ratio	0.739
Total Number Positive Nodes	0.702
N0/N+	0.661
ENE	0.637
Treatment	0.372
LVI	0.309
Differentiation	0.124
PNI	0.095
Margins	0.061
**MYELOID^{MV}**	**squared loading**
WBC	0.91
Neutrophils	0.85
Monocytes	0.39
Sex	0.16
**LYMPHOID^{MV}**	**squared loading**
PLR	0.85
NLR	0.63
Lymphocytes	0.55
PLT	0.14
**TUMOR^{MV}**	**squared loading**
Bone invasion	0.99
pT VIII	0.99
**Gain in cohesion (%)**	**46.85**

**IT-CD8^{MV}**	**loading**
CD8 density Total	0.9866191
CD8 density CT	0.9286171
CD8 density IM	0.9237935
Age	0.3117396
**NODAL^{MV}**	**loading**
Nodal ratio	0.8597471
Total Number Positive Nodes	0.8376385
Differentiation=G1	-0.83438891
pN VIII=pN0	-0.77263130
N0/N+=pN0	-0.76093557
Treatment=Surgery	-0.64286893
ENE=No	-0.43708058
LVI=No	-0.35172077
PNI=No	-0.30449926
Margins=Negative	-0.14653372
Differentiation=G2	-0.12520442
Margins=Close	-0.05056247
pN VIII=pN1	0.01399685
Treatment=Surgery+RT	0.20589926
PNI=Yes	0.31126592
Differentiation=G3	0.33651668
pN VIII=pN2a	0.44018873
Margins=Positive	0.52492533
pN VIII=pN2b	0.65695459
pN VIII=pN2c	0.85813671
N0/N+=pN+	0.86836177
Treatment=Surgery+CRT	0.87404470
LVI=Yes	0.87930191
ENE=Yes	1.4569353
pN VIII=pN3b	1.5517895
**MYELOID^{MV}**	**loading**
WBC	0.9548163
Neutrophils	0.9196095
Monocytes	0.6244274
Sex=Female	-0.5241825
Sex=Male	0.3053933
**LYMPHOID^{MV}**	**loading**
PLR	0.9242590
NLR	0.7958679
Lymphocytes	-0.7388330
PLT	0.3775898
**TUMOR^{MV}**	**loading**
pT VIII=pT2	-0.4853636
pT VIII=pT1	-0.4853636
Bone invasion=No	-0.4769100
pT VIII=pT3	-0.4558341
Bone invasion=Cortical	1.9893744
pT VIII=pT4	2.1154294
Bone invasion=Medullary	2.1529270

cells

MDPI

Supplementary Table S11: Extensive details of multivariable survival Cox proportional hazards models. Legend: d.f., degrees of freedom; Coefficient, regression coefficient; S.E., standard error.

**Overall Survival (OS) - Wald statistics**				**Overall Survival (OS) - Model Details**		
**Variable**	**Chi-Square**	**d.f.**	**P**	**Term**	**Coefficient**	**S.E.**
MYELOID^{MV}	3.09	1	0.0786	MYELOID^{MV}	0.1322	0.0752
NODAL^{MV}	34.00	3	<.0001	NODAL^{MV}	-0.3837	0.1320
Nonlinear	0.63	2	0.7306	NODAL$^{MV'}$	0.2290	0.2896
IT-CD8^{MV}	1.06	1	0.3022	NODAL$^{MV''}$	-17.252	22.966
TUMOR^{MV}	1.44	1	0.2303	IT-CD8^{MV}	-0.0800	0.0775
LYMPHOID^{MV}	10.26	3	0.0165	TUMOR^{MV}	-0.0916	0.0763
Nonlinear	10.25	2	0.0059	LYMPHOID^{MV}	-0.9900	0.3169
**TOTAL NONLINEAR**	**10.42**	**4**	**0.0340**	LYMPHOID$^{MV'}$	46.362	15.525
**TOTAL**	**41.31**	**9**	**<.0001**	LYMPHOID$^{MV''}$	-132.165	47.542

**Disease specific survival (DSS) - Wald statistics**				**Disease specific survival (DSS) - Model Details**		
**Variable**	**Chi-Square**	**d.f.**	**P**	**Term**	**Coefficient**	**S.E.**
MYELOID^{MV}	6.26	1	0.0123	MYELOID^{MV}	0.2389	0.0955
NODAL^{MV}	40.90	3	<.0001	NODAL^{MV}	-0.4373	0.1444
Nonlinear	1.01	2	0.6024	NODAL$^{MV'}$	0.2302	0.3432
IT-CD8^{MV}	0.72	1	0.3956	NODAL$^{MV''}$	-26.953	29.459
TUMOR^{MV}	1.93	1	0.1651	IT-CD8^{MV}	-0.0766	0.0902
LYMPHOID^{MV}	7.74	3	0.0516	TUMOR^{MV}	-0.1247	0.0898
Nonlinear	7.64	2	0.0220	LYMPHOID^{MV}	-0.9734	0.3971
**TOTAL NONLINEAR**	**8.34**	**4**	**0.0800**	LYMPHOID$^{MV'}$	52.910	19.297
**TOTAL**	**48.11**	**9**	**<.0001**	LYMPHOID$^{MV''}$	-166.010	60.157

**Locoregional recurrence free survival (LRRFS) - Wald statistics**				**Locoregional recurrence free survival (LRRFS) - Model Details**		
**Variable**	**Chi-Square**	**d.f.**	**P**	**Term**	**Coefficient**	**S.E.**
MYELOID^{MV}	11.69	3	0.0085	MYELOID^{MV}	0.8172	0.4655
Nonlinear	2.87	2	0.2384	MYELOID$^{MV'}$	-14.469	0.9600
NODAL^{MV}	20.42	3	0.0001	MYELOID$^{MV''}$	70.322	42.615
Nonlinear	5.03	2	0.0809	NODAL^{MV}	-0.3942	0.1565
IT-CD8^{MV}	0.01	1	0.9179	NODAL$^{MV'}$	0.5845	0.3373
TUMOR^{MV}	4.58	1	0.0323	NODAL$^{MV''}$	-59.482	27.493
LYMPHOID^{MV}	0.31	1	0.5758	IT-CD8^{MV}	-0.0080	0.0772
**TOTAL NONLINEAR**	**7.24**	**4**	**0.1240**	TUMOR^{MV}	-0.1742	0.0814
**TOTAL**	**32.98**	**9**	**0.0001**	LYMPHOID^{MV}	0.0536	0.0958

**Distant recurrence free survival (DRFS) - Wald statistics**				**Distant recurrence free survival (DRFS) - Model Details**		
**Variable**	**Chi-Square**	**d.f.**	**P**	**Term**	**Coef**	**S.E.**
MYELOID^{MV}	2.78	1	0.0957	MYELOID^{MV}	0.2005	0.1203
NODAL^{MV}	31.40	3	<.0001	NODAL^{MV}	-0.6192	0.2106
Nonlinear	0.69	2	0.7080	NODAL$^{MV'}$	0.3937	0.5101
IT-CD8^{MV}	9.62	3	0.0221	NODAL$^{MV''}$	-37.339	44.941
Nonlinear	5.85	2	0.0537	IT-CD8^{MV}	-24.305	10.371
TUMOR^{MV}	0.07	1	0.7944	IT-CD8$^{MV'}$	212.985	127.036
LYMPHOID^{MV}	11.92	3	0.0077	IT-CD8$^{MV''}$	-370.310	233.995
Nonlinear	11.22	2	0.0037	TUMOR^{MV}	0.0381	0.1462
**TOTAL NONLINEAR**	**17.74**	**6**	**0.0069**	LYMPHOID^{MV}	-17.050	0.4968
**TOTAL**	**41.19**	**11**	**<.0001**	LYMPHOID$^{MV'}$	86.064	26.509
				LYMPHOID$^{MV''}$	-257.221	83.674
